# Therapeutic efficacy of repetitive transcranial magnetic stimulation in an animal model of Alzheimer’s disease

**DOI:** 10.1038/s41598-020-80147-x

**Published:** 2021-01-11

**Authors:** Jin Seung Choung, Jong Moon Kim, Myoung-Hwan Ko, Dong Sik Cho, MinYoung Kim

**Affiliations:** 1 Rehabilitation and Regeneration Research Center, CHA University, Seongnam, Republic of Korea; 2 Department of Rehabilitation Medicine, CHA Bundang Medical Center, CHA University, 59 Yatap-ro, Bundang-gu, Seongnam, Gyeonggi-do 13496 Republic of Korea; 3 Department of Physical Medicine and Rehabilitation, Jeonbuk National University Medical School, Jeonju, Republic of Korea; 4 R&D Center, Remed Co., Ltd., Seongnam, Republic of Korea

**Keywords:** Neuroscience, Neurology

## Abstract

Previous studies on repetitive transcranial magnetic stimulation (rTMS) suggested potential neurorestorative properties in Alzheimer’s disease (AD). This study aimed to investigate therapeutic effects of rTMS on an AD mouse model at high and low frequencies. The subject mice were allocated into the AD model group (AD induced by intracerebroventricular amyloid beta 42 oligomer [Aβ42] injection) and the saline-injected control group. Each group was subdivided according to rTMS treatment: high frequency (20 Hz), low frequency (1 Hz), and not rTMS-treated. Behavioural assessments with Y-maze test and novel object recognition task were performed; the results indicated cognition recovery by both the frequencies of rTMS after treatment in the AD model (*Ps* < 0.01). Tendency of further effects by high frequency compared to low frequency rTMS was also shown in Y-maze test. Neurotransmitter assay showed increment in dopamine concentration and upregulation of dopamine-receptor 4 (DR4) by rTMS in AD mice with higher response by high frequency stimulation (*Ps* < 0.05). Only high-frequency rTMS induced an elevation of brain-derived neurotrophic factor (BDNF) levels and enhanced the expression of Nestin and NeuN in the brain tissue (*Ps* < 0.05). Under in vitro conditions, Aβ42 incubated mouse hippocampal cell showed an increase in dopamine levels and BDNF by application of high-frequency rTMS treatment. In conclusion, rTMS might have a potential therapeutic effect on AD, and it seems to be related with dopaminergic activation. High frequency of stimulation seems to induce higher efficacy than that induced by low frequency, with elevated expressions of DR4 gene and neurogenic proteins.

## Introduction

Alzheimer's disease (AD) is a neurodegenerative disease that results in a loss of cognitive functions, such as memory, attention, perception, language, and executive function^1^. Amyloid beta (Aβ) deposits and hyperphosphorylated tau proteins were known to be implicated in the major pathogenesis of AD by causing pre-and post-synaptic dysfunction and neuronal loss^2,3^. In addition, the cortical plasticity of long-term potentiation (LTP) was found to be disrupted in AD patients^4,5^ in whom this was suggested to be a significant predictor of cognitive decline^6^. As AD progresses, the reduction of neurotrophic factors and the imbalance of neurotransmitters cause cognitive impairment^7,8^. Even though it is well acknowledged that AD is a worldwide health problem, with the increasing longevity, which involves considerable familial and social costs, a clear solution has not been suggested up to date^9^.

In the recent years, the use of repetitive transcranial magnetic stimulation (rTMS) in diseases with cognitive impairment has shown neurorestorative effects with increasing numbers^10,11^. According to previous AD animal experiments, rTMS reverses Aβ42-induced depletion of nerve growth factor and brain-derived neurotrophic factor (BDNF) in the hippocampus, increases BDNF binding affinity for TrkB in the prefrontal cortex, enhances hippocampal LTP, and reduces Aβ precursor proteins in the hippocampus^12–15^. Neurotransmitters are reportedly involved in AD, and dopaminergic dysfunction has a pathogenic role in aggravation of this symptom^16,17^. The rTMS may affect the dopaminergic pathway to trigger its activation with the incremental release of dopamine in hippocampal cells and receptor dependent neuronal substrate changes within the stimulated area; however, this has not been proved in AD model^18,19^. Clinical trials investigating rTMS showed improvements in spatial working memory function, naming, and verbal memory, possibly through its action via the previously described mechanisms^20–23^.

The rTMS generates high intensity electrical pulses using a magnetic coil, and rTMS protocols may vary in intensity, duration, and frequency^13,24,25^. In the application of rTMS, factors in the protocol might influence the neural cell mechanics and frequency of stimulation is one of the main factors. Both high and low frequency rTMS were suggested to alter the excitatory status of cortical cells with comparable mode of action^10^. However only one clinical study compared high- and low-frequency rTMS treatment in AD patients; this study showed better outcomes with high-frequency than low-frequency stimulation^26^. There have been two animal studies comparing frequency; however, these are limited as references for clinical application because of their short stimulation durations and short total stimulation days^14,27^, which are quite different from the usual rTMS protocols in AD clinical trials^23,28^. To better inform clinical application, an evaluation of the methods of cognitive function in AD model animals is also important. So far, cognitive behavioural assessments are the most objective tool, and the Y-maze test and Novel Object Recognition Task (NORT) are useful at determining neurocognitive measurements in rodent models. The Y-maze test assesses the natural behaviour of rodents to explore new environments according to their memory and NORT also investigates their memory function by analysing their ability to recognize novel objects^29,30^.

**Figure 1:**
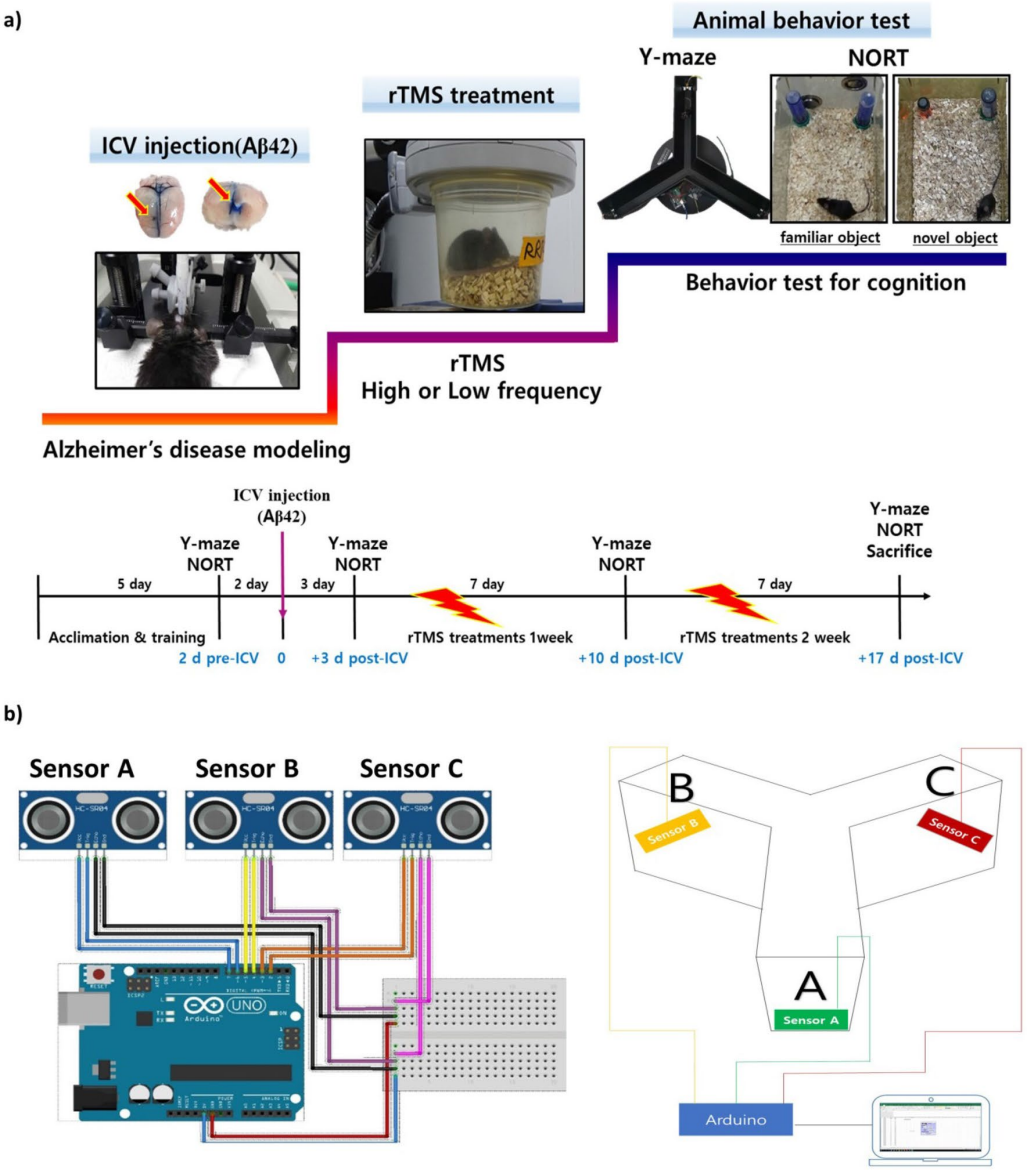
Experimental schematic and schedules. (**a**) The mice were administered Aβ42 oligomer via ICV using stereotaxic instrument with acceptable injection range (− 0.9 mm posterior, 1.7 mm right lateral, and 2.2 mm depth from bregma). Top view of the whole brain and coronal section show bilateral lateral ventricles filled with trypan blue. Two days before and three days after the Aβ42 injection, Y-maze test and NORT were performed to determine whether the Aβ42 induced Alzheimer’s disease status. After ICV administration, experimental groups were treated rTMS for 2 weeks while the control group did not receive rTMS. At the end of each week of intervention, Y-maze test and NORT were repeated. (**b**) Design and picture of an automatic Y-maze test system with an ARDUINO. Wiring diagram was drawn using FRITZING.ORG (developed by Friends-of-Fritzing, Germany) for the Arduino UNO microcontroller connected to three sonar sensors programmed to sensing either distance or location. *Aβ42* amyloid beta 42 oligomer, *ICV* intracerebroventricular injection, *NORT* novel object recognition task, *rTMS* repetitive transcranial magnetic stimulation.

In this study, the therapeutic effects of high and low frequency rTMS treatments were evaluated with the cognitive motor behaviour tests, using a mouse model of AD induced by intracerebroventricular (ICV) Aβ42 oligomer injection (Fig. 1). To assess the therapeutic actions of rTMS in the brain tissue, levels of Aβ, neurotransmitters, neurotrophin, and markers of neurogenesis were measured and analysed by comparing effects among the different treated groups. In vitro experiment was also conducted to verify direct effect of rTMS on neuronal cells.

## Results

### rTMS treatment enhanced spatial working memory in the Y-maze test and NORT in AD mouse

To determine whether the spatial working memory reduced by Aβ42 administration into the brain, spontaneous alternation (SA) rate of Y-maze test and recognition Index (RI) of NORT were measured in this experiment^29,30^. The values of SA rate and RI did not differ between the AD group and the phosphate-buffered saline (PBS) group before the procedure but decreased in AD group at 3 days after Aβ42 injection inducing AD pathogenesis. The rTMS treatment of frequencies, high or low were administered 3 days after Aβ42 injection for 2 consecutive weeks, 5 days a week.

**Figure 2:**
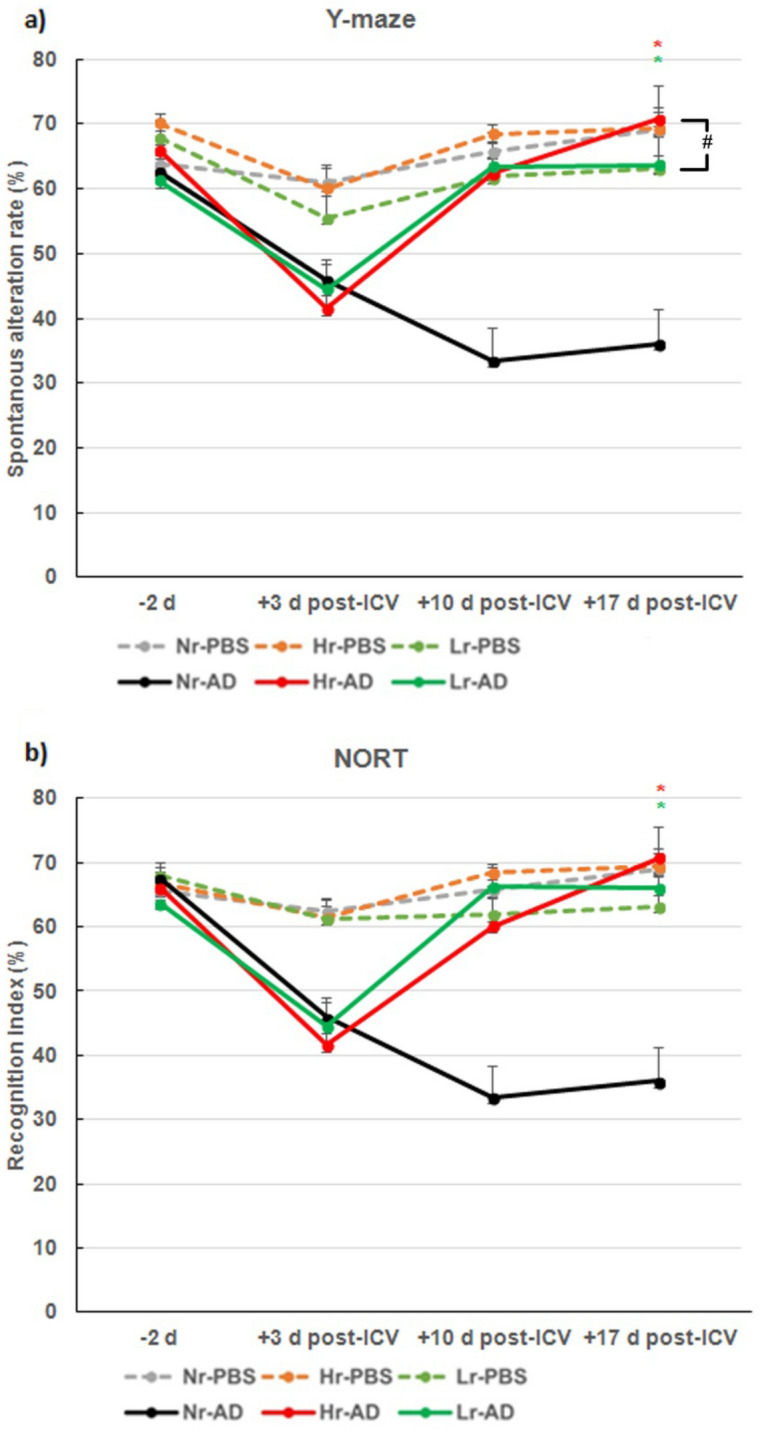
rTMS treatment up-regulates spatial working memory in the Y-maze test and recognition index of NORT in AD mouse model. (**a**) Spontaneous alternation rate over time by Y-maze test. (**b**) Recognition index over time by NORT. Each group n = 8, mean ± SEM. *Ps < 0.05; Hr-AD and Lr-AD compared to those in Nr-AD, ^#^*P* = *0.06,* Hr-AD compared to Lr-AD (Y-maze, F (2, 42) = 14.620, NORT, F (2, 42) = 11.017). *rTMS* repetitive transcranial magnetic stimulation, *NORT* novel object recognition task, *AD* Alzheimer’s disease model, *ICV* intracerebroventricular injection, *NR−* rTMS not treated, *Hr*− high frequency rTMS treated, *Lr−* low frequency rTMS treated, *PBS* Phosphate-Buffered Saline.

In the AD group, the SA rates and RI increased in both high-frequency rTMS-treated subgroup (Hr-AD) and low-frequency rTMS-treated subgroup (Lr-AD) compared to those in none rTMS treated subgroup (Nr-AD) (n = 8 in each subgroup) by 3 × 3 × 2 ANOVA that analysed for time, group (PBS and AD), and rTMS treatment (none, high-frequency, and low-frequency) (Y-maze, F (2, 42) = 14.620, Ps < 0.001, NORT, F (2, 42) = 11.017, Ps < 0.001) (Fig. 2). There were overall differences in SA rate and RI at the different time points (Ps < 0.01). When compared differences between Hr-AD and Lr-AD, Hr-AD showed higher elevation of SA rate than Lr-AD with marginal significance (P = 0.06), and it did not show difference in RI between the groups.

### rTMS increases dopamine concentration and expression of dopamine receptor 4 in AD mouse

**Figure 3:**
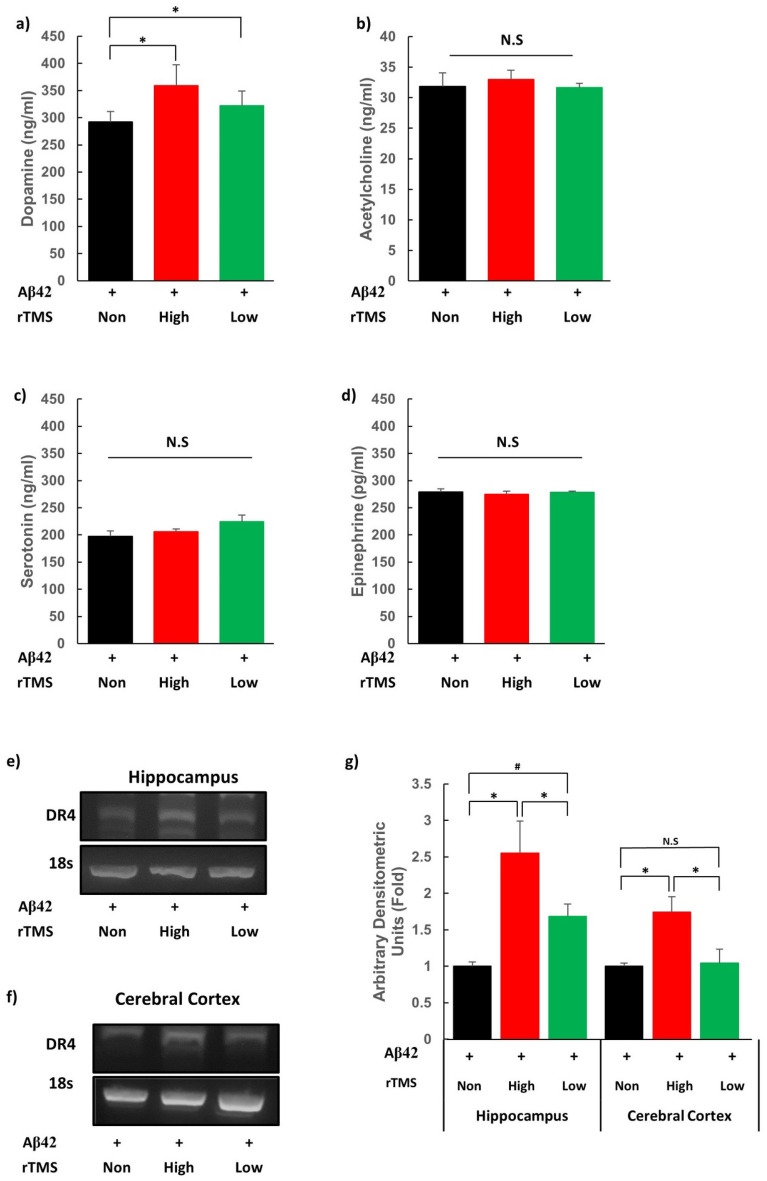
rTMS elevates concentration of dopamine and gene expression of DR4 in AD mouse model. The concentrations of neurotransmitters were measured after rTMS from tissue homogenates lysate of the hippocampus (**a**) Dopamine, (**b**) Acetylcholine, (**c**) Serotonin, and (**d**) Epinephrine. rTMS regulated dopaminergic signalling pathway through DR4. The expression of DR4 was measured by RT-PCR in (**e**) Hippocampus and (**f**) Cortex (**g**) Histograms show densitometry analysis of DR4 RT-PCR. Each group n = 3, mean ± SEM.*Ps < 0.05, ^#^ = 0.069. *+* Aβ42 oligomer injected via ICV, *rTMS* repetitive transcranial magnetic stimulation, *DR4* Dopamine receptor 4, *AD* Alzheimer’s Disease, *RT-PCR* reverse transcription polymerase chain reaction, *Aβ42* amyloid beta 42 oligomer, *None* none treatment, *High* high frequency rTMS treated, *Low* low frequency rTMS treated, *N.S.* not significant.

AD is characterised by markedly reduced concentration of neurotransmitters such as acetylcholine and glutamate in hippocampus and neocortex^31^. Concentrations of dopamine, acetylcholine, serotonin, and epinephrine and were measured in the brain tissue of the AD mouse in the hippocampus, cerebral cortex, and cerebellum after dissection after the 2-week treatment time point. Significant difference in concentration of neurotransmitters was observed only at the hippocampus with higher levels of dopamine in Hr-AD and Lr-AD compared to Nr-AD (Ps < 0.05) (Fig. 3a-d). Gene expression of dopamine receptor subtypes (dopamine receptor 1, 2, 3, 4, and 5) were identified by reverse transcription polymerase chain reaction (RT-PCR). The level of dopamine receptor 4 (DR4) mRNA was higher in Hr-AD compared to Nr-AD and Lr-AD (*Ps* < 0.05), while it was marginally higher in Lr-AD as compared to Nr-AD (*P* = 0.069) at the hippocampus. The level increased only in Hr-AD as compared to Nr-AD and Lr-AD (*Ps* < 0.05) at the cerebral cortex (Fig. 3e-g). The therapeutic efficacy of rTMS on AD might be related to upregulations of dopamine level and DR4 expression by both frequencies of stimulation. Meanwhile, higher DR4 gene expression in Hr-AD brain tissues might be associated with better neurocognitive progress during 2 weeks of treatment induced by high frequency stimulation.

### rTMS increased neurogenesis in AD mouse model

**Figure 4:**
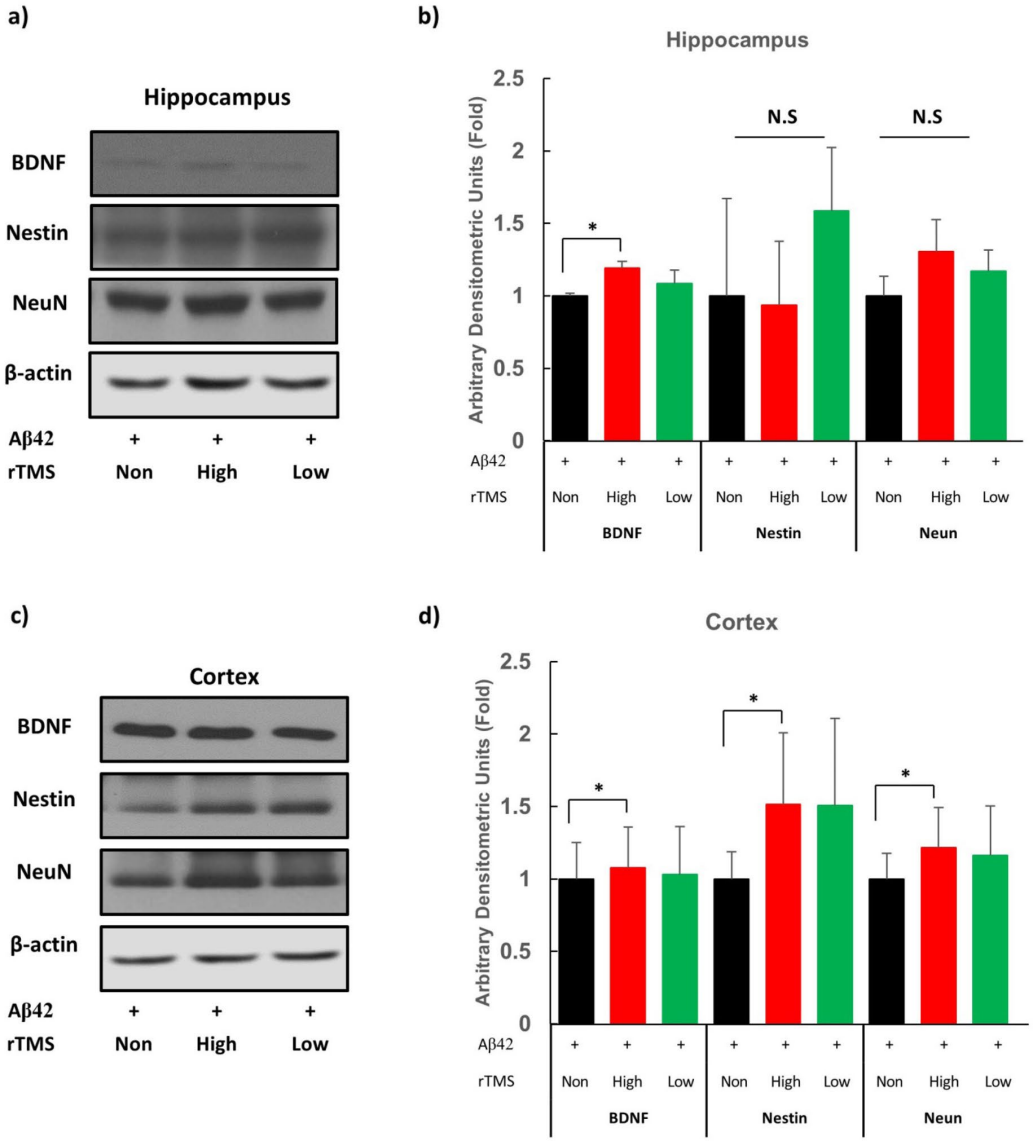
rTMS upregulates neurogenic expressions in AD mouse model. (**a**) Protein expression of hippocampal BDNF, Nestin and NeuN protein was measured by western blot. (**b**) Histograms show densitometry analysis of the western blot in hippocampus. (**c**) Protein expression of cortex BDNF, Nestin and NeuN protein was determined by the western blot. (**d**) Histograms show densitometry analysis of the western blot in cerebral cortex. Protein level of actin was analysed as a loading control. Mean data normalised to β-actin are in bar graphs compared with control. Each group n = 4, mean ± SEM.*Ps < 0.05. *+* Aβ42 oligomer injected via ICV, *rTMS* repetitive transcranial magnetic stimulation, *AD* Alzheimer’s Disease, *BDNF* brain-derived neurotrophic factor, *Aβ42* amyloid beta 42 oligomer, *None* none treatment, *High* high frequency rTMS treated, *Low* low frequency rTMS treated, *N.S.* not significant.

To examine the effects of rTMS on neurogenesis in the AD mouse after the 2-week treatment, major markers of neurogenesis, such as BDNF, nestin, and NeuN were assayed using western blotting technique^32^. The protein expression level of BDNF was significantly higher only in Hr-AD as compared to Nr-AD at the hippocampus and cerebral cortex (*Ps* < 0.05) (Fig. 4a–c). The levels of nestin and NeuN were also higher in Hr-AD as compared to Nr-AD at cerebral cortex tissue (*Ps* < 0.05) (Fig. 4c,d). These results indicate that rTMS exerts neurogenic and neuroprotective effects.

### Repetitive magnetic stimulation (rMS) down-regulates the expression levels of amyloid beta and upregulates expression of dopamine receptor 4 (DR4) in vitro

To confirm direct effects of repetitive magnetic stimulation on neuronal cells with AD, an in vitro experiment was performed. This was done by incubating HT-22 cells (mouse hippocampal cell line) with Aβ42 to mimic the AD models in vitro.

**Figure 5:**
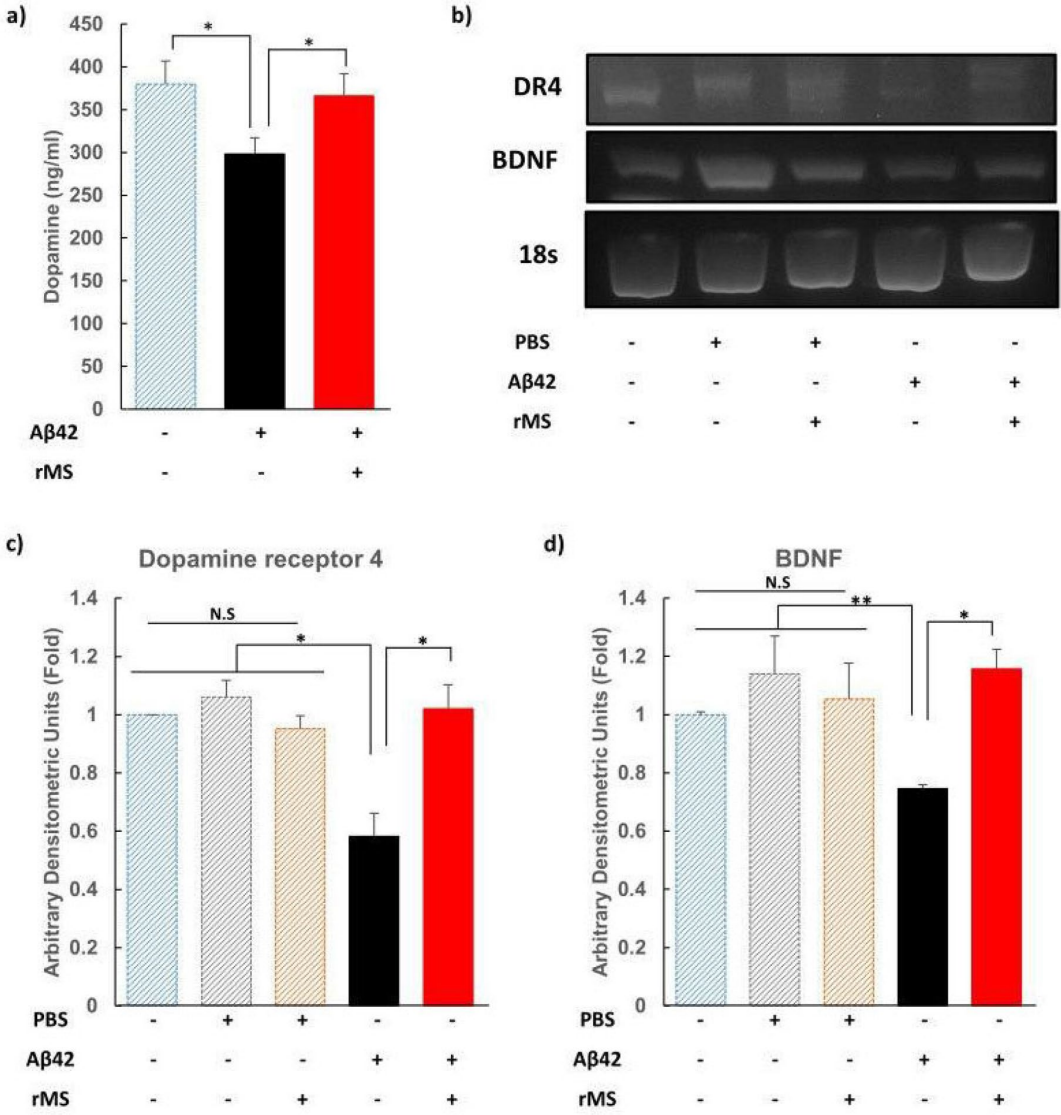
rMS increased expression of DR4 in vitro HT-22. The Aβ42 was added to the hippocampal cell line of mouse (20 μM Aβ42 was treated at 24 h in the serum free condition in specifically HT-22 cells). (**a**) Dopamine levels were measured by ELISA. (**b**) Gene expression of DR4 and BDNF was measured by RT-PCR. Densitometry analysis of RT-PCR (**c**) DR4, (**d**) BDNF. Data are shown as the DR4 or BDNF/18 s ratio. The mRNA level of 18 s was analysed as an internal control. Each group n = 4, mean ± SEM. *Ps < 0.05, **Ps < 0.01. *+* treatment, *−* none treatment of PBS/Aβ42 oligomer/rMS, *rMS* repetitive magnetic stimulation with 20 Hz frequency, *DR4* Dopamine receptor 4, *Aβ42* amyloid beta 42 oligomer, *ELISA* Enzyme-Linked Immunosorbent Assay, *BDNF* brain-derived neurotrophic factor, *RT-PCR* reverse transcription polymerase chain reaction, *PBS* Phosphate-buffered saline, *N.S.* not significant.

The stimulation protocol of rMS consisted of 40 trains stimulation for 2 s at 20 Hz with an inter-train interval of 28 s with a total of 1600 pulses per session with 1.26 T intensity for 3 days. The frequency of magnetic stimulation was selected because expressions of DR4, BDNF, nestin, and NeuN were increased in the brain of Hr-AD in vivo experiments while both of Hr-AD and Lr-AD groups showed therapeutic effects without differences in the behaviour. As results, the concentration of dopamine was lower in the Aβ42 administered AD cells as compared to non-Aβ42 added control cells (*Ps* < 0.05). The concentration of dopamine was higher in high-frequency rMS-treated cells than in those not rMS-treated in the AD model (*Ps* < 0.05) (Fig. 5a). The mRNA levels of DR4 and BDNF were lower in Aβ42-administered cells than in all non-Aβ42 administered cells: control, PBS added without rMS treatment (Nr-PBS), and PBS added with high-frequency rMS (Hr-PBS) (*Ps* < 0.05). The gene expression levels of DR4 and BDNF were higher in high-frequency rMS treated cells as compared to those not rMS-treated cells in the AD model (*Ps* < 0.05) (Fig. 5b–d). Thus, this in vitro assay indicates recovering effect of high-frequency rMS by upregulation of dopamine concentration and DR4 and BDNF gene expressions in the AD model neuronal cells, which were downregulated by Aβ42 administration.

## Discussion

In this study, rTMS showed a remarkable therapeutic potential for AD by demonstrating improvements in cognition-related behaviours with dopaminergic activation and upregulation of neurogenic signals. Although both high and low frequency of rTMS seemed to enhance spatial working memory and sensorimotor capacity, rTMS at high frequency induced greater dopaminergic activation and neurogenic effects. The in vitro results using AD model neuronal cells were in concordance with the in vivo findings.

To measure cognitive function, a hallmark clinical symptom that is deteriorated in AD, we used Y-maze test and NORT as behavioural assessments, which were also used in recent studies^29,30^. The SA rate of Y maze and RI of NORT, also known as the representative indicators of learning and memory ability, revealed successful AD modelling by ICV injection of Aβ42 oligomer in Nr-AD group with lower values compared to those in the all control PBS-injected groups. The therapeutic effect of rTMS could be appreciated by higher values of SA rate and RI in both Hr-AD and Lr-AD groups as compared to those in the Nr-AD group. These results are in concordance with previous results that showed improvement of memory in Morris water test in AD models by both low-frequency (1 Hz) or high-frequency (10 or 15 Hz) rTMS treatments^12,14^. The rTMS treatment at different frequencies may exert different effects; high-frequency stimulation seems to have a higher therapeutic efficacy for AD than does low-frequency stimulation^26^. In a recent clinical study, high frequency rTMS to the precuneus induced a selective improvement in episodic memory with increases of neural activity in patients’ precuneus and modification of functional connections between the precuneus and medial frontal areas^33^. In the present in vivo behavioural study, both high and low frequencies of rTMS treatments showed therapeutic efficacy in AD model. And a tendency of further improvement was found by high frequency rTMS compared to low frequency in SA rate.

On analysing the therapeutic mechanisms of rTMS for AD, we could observe dopaminergic activation and possible enhancement of neurogenesis. Amongst the assessed neurotransmitters, dopamine overpowered the rest and was found to be elevated in the hippocampus using both frequencies, high and low, of the rTMS treatment. Amongst its receptors, only DR4 expression was enhanced at the hippocampus and cerebral cortex by both frequencies of rTMS, but a greater enhancement was observed by high-frequency than low-frequency stimulation at the hippocampus, which was significant. According to previous researches that showed importance of dopaminergic transmission in recovering from AD, administration of dopamine receptor agonist induced degradation of Aβ and restored LTP-like cortical plasticity^34,35^. The DR4 identified in this study was reported to have a role in working memory performance^36^, and had also a restoring effect in LTP at hippocampus in the aged mice^37^.

As for enhancing neurogenesis, we observed increments in expression of BDNF, nestin, and NeuN at the cerebral cortex and BDNF at the hippocampus by only high-frequency rTMS. The BDNF is one of the most important neurotrophic factors causing synaptic plasticity by the BDNF-TrkB system. BDNF–TrkB signalling regulates multiple brain functions, such as differentiation, neuronal survival, and neurogenesis^38^. The Nestin and NeuN are expressed in different neuronal differentiation stages; however, elevation of both markers can be interpreted as enhanced neurogenic activity in this experiment^32^.

Although there is a limitation confirmed only in high frequency rTMS, the results of in vivo study could be reproduced in HT-22 cell line in vitro with an increment of dopamine concentration and gene expressions of DR4 and BDNF by magnetic stimulation. Therefore, rTMS seemed to activate the dopaminergic pathway and enhance neurogenicity in vivo*,* as demonstrated by the responses from neuronal cells in the hippocampus and cortex.

According to the results of present study, rTMS at different frequencies may exert different effects and high-frequency (20 Hz) stimulation seems to exert a higher effect in the brain of AD than does low-frequency (1 Hz) stimulation. While both high and low frequency rTMS resulted in similar responses in the main cognitive behaviours with similar levels of DR4 gene expression, only high frequency rTMS brought greater expressions in DR4 gene in the hippocampus and cerebral cortex (Fig. 3). Moreover, only high frequency rTMS induced higher neurogenic protein (BDNF, Nestin, and NeuN) expressions in the cortex and BDNF expression in the hippocampus (Fig. 4). Clinical studies of rTMS showed dopamine release induced by most high-frequency stimulation rather than low-frequency stimulation^19,39–41^. Elevation of BDNF expression seems to be significant, as it plays an important role in enhancing spatial working memory in the hippocampus through activation of BDNF-mediated NMDA receptors^42^.

There are some limitations to consider in this study. The experimental animal model in this study was not a transgenic mouse model; thus, the pathophysiology and therapeutics cannot fully reflect an actual AD patient’s brain. The long-term effects could not be confirmed because of the limitations related to the model. Covariant factors that could alter the effects of rTMS, such as APOE polymorphism, were not controlled^43^. Since APOE polymorphism has been recently shown to have an impact on rTMS effects in AD patients, further studies investigating the interplay between the dopaminergic system, plasticity and APOE are required. The stimulation protocol used in this study included covering the whole brain using a coil (non-specific); thus, the results cannot be generalised to the human brain The total levels of BDNF, nestin, NeuN, and dopamine increased but the direct therapeutic mechanism and effect on synaptic plasticity were not identified. And the important part of dopaminergic transmission, the target part of the ventral tegmental area, was not identified by separating the prefrontal cortex. Further studies are required to clarify therapeutic mechanisms of rTMS and ideal stimulation conditions through localized brain region analysis.

## Conclusion

The rTMS in the AD mouse model showed a recovering effect in neurobehavioral test concurrent with an activation of dopaminergic pathway and neurogenesis. The high-frequency stimulation seemed to exert neurogenic effect in the brain tissue.

## Materials and methods

### Animals

All animal experiments were carried out under a protocol approved by the Institutional Animal Care and Use committee at Cha University (IACUC180061). C57BL/6 male mice (8 weeks old) were purchased from Orient Bio Inc. (Seoul, Republic of Korea), maintained at a facility at Cha University, and habituated for 7 days. During the procedure, the mouse was positioned on a heating pad to maintain normothermia. Isoflurane was administered with the use of a VEVO COMPACT ANESTHESIA SYSTEM. Isoflurane induction was performed by the same individual every time, with the nose of the mouse placed into a small nose cone using 3% isoflurane in pure medical oxygen. Then, Aβ42 (10 μM) in phosphate-buffered saline (PBS) (10% dimethyl sulfoxide [DMSO]) was incubated at 37 °C for 1 week to obtain soluble oligomeric species, and then, 5 μl of Aβ oligomer^44^ or vehicle (90% PBS and 10% DMSO) was acutely injected (0.5 µL/min) into the intracerebroventricular space in the lateral ventricle. The injection site was determined according to the stereotaxic atlas^45^ (− 0.9 mm posterior, 1.7 mm right lateral, and 2.2 mm depth from bregma)^44^.

To establish an appropriate model for Aβ42 ICV injection, a preliminary practice was repeatedly conducted using trypan blue prior to the experiment. After the injection, any neurological signs representing brain damage were observed, and the brain structure was confirmed with distribution of the dye following extraction and dissection (Fig. 1). The experiments started after achieving a success rate of > 95%, which is usually achieved after more than 100 ICV injections^44^; this was similar to the experiences of 110 ICV injections in the present study. Determination of dementia modeling was executed 3 days after the ICV injection by use of cognitive behavioral assessments. Individuals whose SA rate and RI rate did not decrease below 45% were excluded from the experiment. Consequently, in this study, 48 out of a total of 60 animals were selected and examined.

### Cell culture

Cell cultures were generated using immortalised mouse hippocampus HT-22 cells, purchased from the ATCC (Manassas, VA, USA). HT-22 was cultured in Dulbecco's Modified Eagle medium supplemented with 10% (v/v) heat-inactivated foetal bovine serum, 25 mM glucose, 2 mM l-glutamine, 100 U/mL penicillin, and 100 μg/mL streptomycin at 37 °C in a humidified atmosphere containing 95% (v/v) air and 5% (v/v) CO_2_. The cells were seeded at a density of 2.0 × 10^5^ cell/well in 6-well plates. To induce the Alzheimer’s in vitro model, 20 μM Aβ42 was treated at 24 h in the serum-free condition. And then HT-22 cells were treated with 3 days of rMS stimulation for 20 min daily. The control group was incubated for 72 h.

### Experimental design

In order to obtain the baseline data, Y maze test and NORT were performed 2 days prior and 3 days after injecting Aβ42 oligomer in the mouse. Experimental groups received rTMS for 2 weeks and for 5 consecutive days in a week. Both behavioural tests, Y-maze and NORT, were performed at the end of each week of intervention. After completion of the last test, the subjects were sacrificed. Approximately, 500 μl of blood per mouse was collected from the abdominal vein, after which the brain was immediately frozen in liquid nitrogen (Fig. 1a).

### Application of rTMS

The rTMS was administered using a round coil (inner diameter 5 cm; outer diameter 7 cm; REMED Company, Republic of Korea). A total of 1600 pulses per session with 1.26 T intensity were used. High frequency rTMS consisted of 40 trains of 2 s duration and at 20 Hz, with an inter-train interval of 28 s. The low frequency rTMS consisted of a continuous 1-Hz stimulation. The coil was placed on the box containing the mouse, and the intensity was confirmed by a gauss meter just over the head of the mouse. Each stimulation was given with confirmation of brain target stimulation when the mouse was not moving. The rTMS did not induce seizure, signs of discomfort, or any apparent behavioural changes except for occasional muscle twitching during rTMS application.

### Behaviour tests

#### Y-maze test

This was used to assess spatial working memory (by SA) of the mouse when placed into a Y-maze. Y-maze is a three-arm horizontal maze (35 cm long and 10 cm wide with 12 cm deep) in which the arms are symmetrically disposed at 120° angles from each other (JeungDo Bio & Plant, Republic of Korea). Each mouse was placed in one of the arm compartments and was allowed to move freely until its tail completely entered another arm. The sequence of arm entries was automatically recorded by ARDUINO, which are sonar sensors providing input to the Y-maze. ARDUINO is an open-source prototyping platform that can be applied to analyse arm entry sequence^46^. ARDUINO expansion board has 14 digital signal and 6 analogue signal interfaces. The board can connect to 20 different sensors at a time. In our tool, we only used three sonar sensors each (Fig. 1b). The mouse was placed at the end of one arm and was allowed to move freely through the maze during 8 min sessions. The number of total arm choices and sequences were recorded. The percent of alternation is defined by proportion of arm choices that differed from the last two choices. Before each trial, the interior of the maze was sprayed with 70% ethanol solution to eliminate any scent cues. The results measured through the ARDUINO were automatically recorded and calculated. As a result, the researchers' prejudice could be excluded, and reliable results were obtained.

#### NORT

This evaluates the recognition memory of a previously explored object (familiar) in comparison with a new object (non-familiar). In order to assess the spatial working memory (recognition index) of the mouse, a mouse trained with a familiar object was freely tested for 5 min, after which one of the two objects was replaced with a new object. The mouse adapted to a familiar object showed interest in the new object, recorded it, and the time it stayed on the new object was calculated. A set of three copies of the same object was used to prevent odor signals, and all combinations and positions of the object were used to prevent preference- or location-based bias. Recognition index was calculated using the following formula: Ratio = $\frac{t1}{t1+t2}$, where t1 = the amount of time mice explored the novel object and t2 = the amount of time mice explored the familiar object.

#### Recording and analysis

Data analyses, including recordings of all behavioural responses, were manually recorded in the computer; the time of mouse stay on object was calculated using a stop watch.

### Neurotransmitter analysis

The sacrificed mouse brain was divided into hippocampus, cortex, midbrain, and hindbrain. Due to differences in the mechanism of action depending on the region of the brain, hippocampus, a typical abnormal region in AD, was isolated from the cortex. Neurotransmitter concentration in brain tissue homogenates were measured by ELISA kit (Acetylcholine: Cell bio labs, Serotonin, Dopamine, and Epinephrine: LS Bio).

Western blotting: Total cell lysates from the mouse brain were prepared using RIPA Protein Extraction Buffer (Sigma) containing a protease and phosphatase inhibitor cocktail (Sigma-Aldrich). The isolated protein (30–50 μg) was electrophoresed in a 10% (w/v) acrylamide–sodium dodecyl sulphate gel and transferred to polyvinylidene difluoride membranes. After transferring from the gel to the membrane, the sizes of the target proteins were checked using a protein ladder to crop the membrane. The membrane was carefully cut using a knife, and then antigen–antibody reactions were conducted after blocking with skim milk according to the protein location, which were purposed to save reagent and to obtain clear image by preventing non-specific bindings. After 1 h of blocking with 5% (w/v) skimmed milk, the membranes were incubated with anti-BDNF (sc-546, Santa Cruz Biotechnology), anti-nestin (ab6142 Abcam), anti-NeuN (ABN78, millipore), or β-actin antibody (sc-47778, Santa Cruz Biotechnology). The membranes were then incubated with horseradish peroxidase-conjugated goat anti-rabbit IgG or goat anti-mouse IgG antibodies. Signals were detected with enhanced chemiluminescence (ECL) detection kit (Millipore) and analysed using a Kodak Scientific Imaging Film (Kodak), and Fixer/Developer and Replenisher (Kodak) according to the manufacturer’s instructions. The films were exposed for 30 s, then developed and dry for 4 hr at room temperature. The weights of the proteins were marked on the film with an oil pen at the same position on the membrane and the film.

RNA isolation and conventional RT-PCR: total RNA was isolated from cells using Trizol according to the manufacturer’s protocol. cDNA was synthesized from 100 ng total RNA using the cDNA synthesis kit (ToYoBo). Conventional PCR was performed using a reaction mixture containing Green master mix (Bio-neer). PCR amplification was carried out in a TAKARA Detection System at 50 °C for 2 min and 95 °C for 10 min, followed by 35 cycles of 95 °C for 15 s and 60 °C for 1 min.

The following specific primers were used:

- BDNF (110 bp): the forward primer was 5′-TGCAGGGGCATAGACAAAAGG-3′; the reverse primer was 5′-CTTATGAATCGCCAGCCAATTCTC-3′.
- DR4 (202 bp) the forward primer was 5′-TGCCCTCAACCCCATCATCTACAC-3′; the reverse primer was 5′-AATACTTCCGACCCCCAACCCT-3′.
- 18 s (150 bp): the forward primer was 5′-GTAACCCGTTGAACCCCATT-3′; the reverse primer was 5′-CCATCCAATCGGTAGTAGCG-3′.

All methods were performed in accordance with the relevant guidelines and regulations (IACUC180061).

### Statistical analysis

The animal’s behavioural activities in Y-maze and NORT were statistically analysed using SPSS version 20.0 (IBM, Chicago, IL, USA). To compare effect by time, group (AD, PBS), and rTMS type in the values of SA rates and RI between subgroups, a 3 × 3 × 2 mixed model analysis of variance (ANOVA) was used. Statistical significance was determined by Tukey’s post hoc test in each comparison between the treatment groups. Since there were significant effects of time on SA rates and RI, further assessments for within group time effects were conducted after testing Mauchly’s assumption of sphericity. Neurotransmitter and densitometry experiments were conducted with a minimum of three different samples, and the data were presented as mean ± standard error. A value of *Ps* < 0.05 was considered statistically significant.

## Supplementary Information

Supplementary Figures.
