# Author Correction: Therapeutic efficacy of repetitive transcranial magnetic stimulation in an animal model of Alzheimer’s disease

**DOI:** 10.1038/s41598-021-86896-7

**Published:** 2021-03-26

**Authors:** Jin Seung Choung, Jong Moon Kim, Myoung-Hwan Ko, Dong Sik Cho, MinYoung Kim

**Affiliations:** 1 Rehabilitation and Regeneration Research Center, CHA University, Seongnam, Republic of Korea; 2 Department of Rehabilitation Medicine, CHA Bundang Medical Center, CHA University, 59 Yatap-ro, Bundang-gu, Seongnam, Gyeonggi-do 13496 Republic of Korea; 3 Department of Physical Medicine and Rehabilitation, Jeonbuk National University Medical School, Jeonju, Republic of Korea; 4 R&D Center, Remed Co., Ltd., Seongnam, Republic of Korea

Correction to: *Scientific Reports* https://doi.org/10.1038/s41598-020-80147-x, published online 11 January 2021

This Article contains an error in Figue 4, where one of the labels is misspelled in panels (b) and (d).

‘Neun’

should read:

‘NeuN’

The correct Figure 4 appears below as Figure 1.

**Figure 1:**
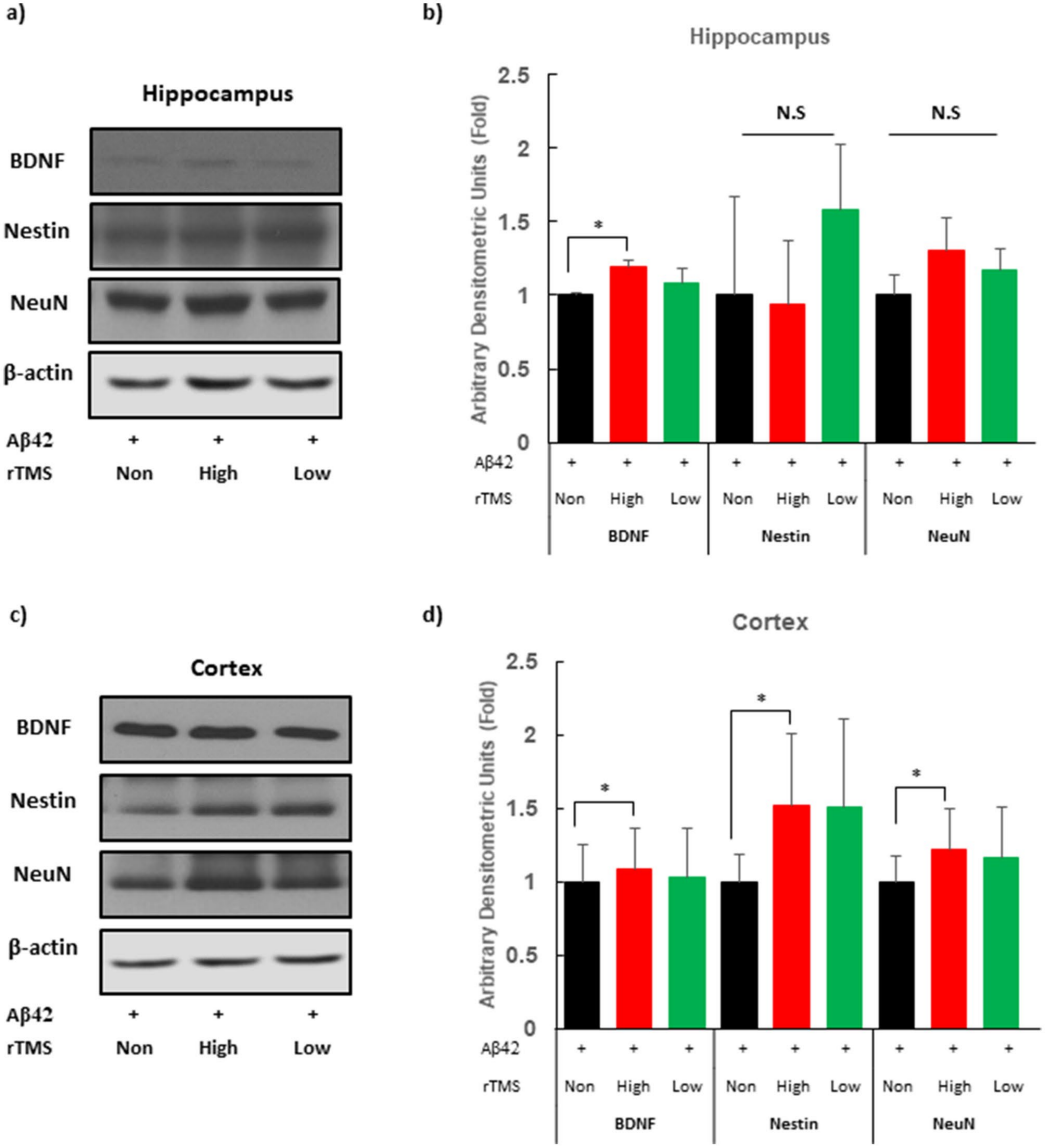
A correct version of the original Figure 4. rTMS upregulates neurogenic expressions in AD mouse model. (**a**) Protein expression of hippocampal BDNF, Nestin and NeuN protein was measured by western blot. (**b**) Histograms show densitometry analysis of the western blot in hippocampus. (**c**) Protein expression of cortex BDNF, Nestin and NeuN protein was determined by the western blot. (**d**) Histograms show densitometry analysis of the western blot in cerebral cortex. Protein level of actin was analysed as a loading control. Mean data normalised to β-actin are in bar graphs compared with control. Each group n = 4, mean ± SEM.* *Ps* < 0.05. + Aβ42 oligomer injected via ICV, *rTMS* repetitive transcranial magnetic stimulation, *AD* Alzheimer’s Disease, *BDNF* brain-derived neurotrophic factor, *Aβ42* amyloid beta 42 oligomer, *None* none treatment, *High* high frequency rTMS treated, *Low* low frequency rTMS treated, *N.S.* not significant.

