# Supplementary material for: Therapeutic efficacy of repetitive transcranial magnetic stimulation in an animal model of Alzheimer’s disease
Source: Sci Rep. 2021 Jan 11;11:437. doi: 10.1038/s41598-020-80147-x (PMC7801521; doi:10.1038/s41598-020-80147-x)
Supplement: Supplementary file 1 — Supplementary Figures. [file 41598_2020_80147_MOESM1_ESM.docx]

**Therapeutic efficacy of repetitive transcranial magnetic stimulation in an animal model of Alzheimer’s disease**

Jin Seung Choung^1^, Jong Moon Kim^1, 2^, Myoung-Hwan Ko^3^, Dong Sik, Cho^4^, and MinYoung Kim^1,2^

*^1^Rehabilitation and Regeneration Research Center, CHA University, Seongnam, Republic of Korea*

*^2^Department of Rehabilitation Medicine, CHA Bundang Medical Center, CHA University, Seongnam, Republic of Korea*

*^3^Department of Physical Medicine and Rehabilitation, Jeonbuk National University Medical School, Republic of Korea.*

*4 R&D Center, Remed Co., Ltd. Seongnam, Republic of Korea*

Jin Seung Choung and Jong Moon Kim contributed equally to this work.

Corresponding author: MinYoung Kim, MD, PhD

Address: Department of Rehabilitation Medicine, CHA Bundang Medical Center,

CHA University, 59 Yatap-ro, Bundang-gu, Seongnam, Gyeonggi-do,

Republic of Korea, 13496

Tel: 82-31-780-1872

Fax: 82-31-780-3449

E-mail: [kmin@cha.ac.kr](mailto:kmin@cha.ac.kr)

**Fig.S1. Supplementary gel data for fig. 3**


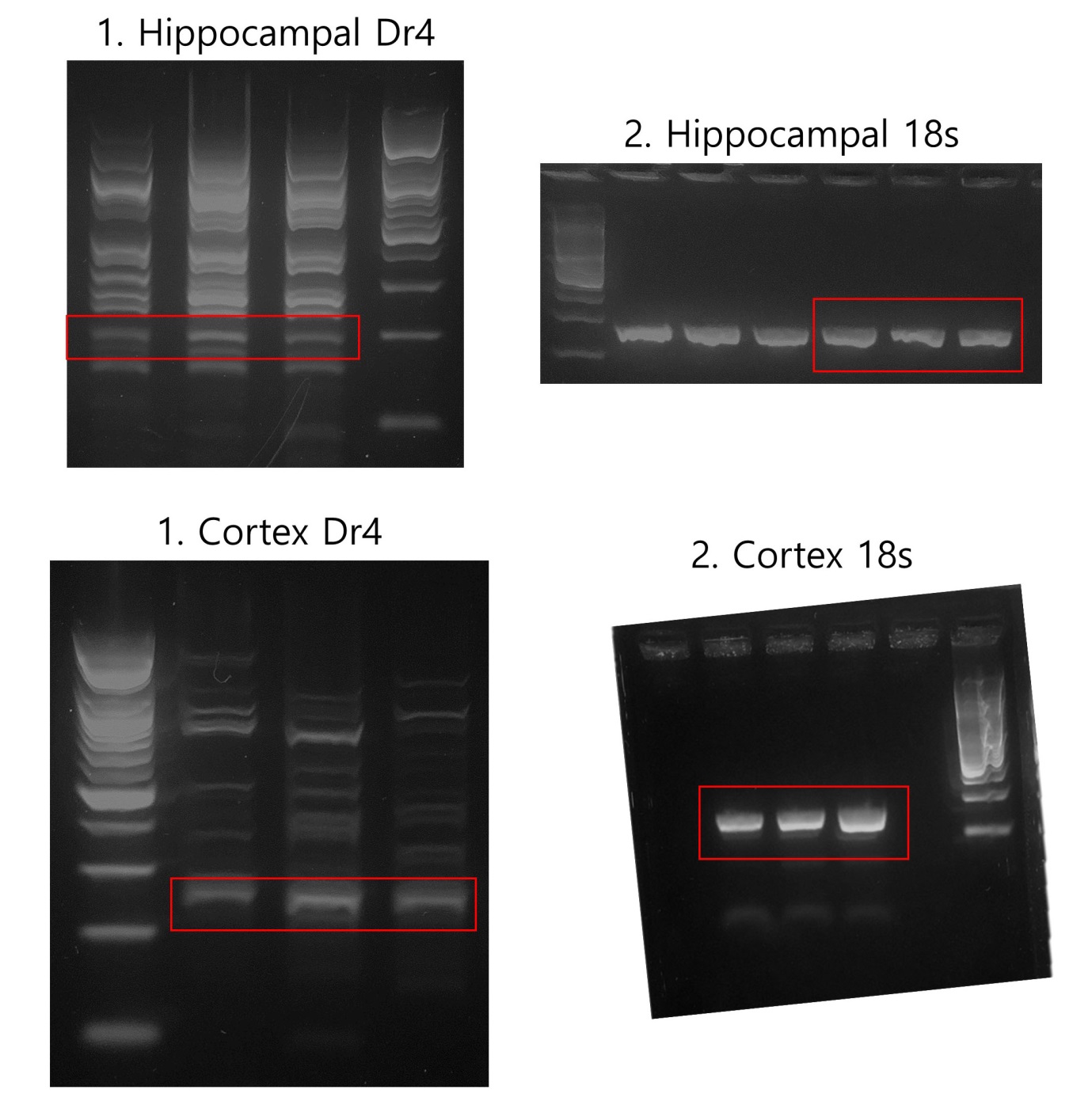


**Fig.S2. Supplementary gel data for fig. 4**


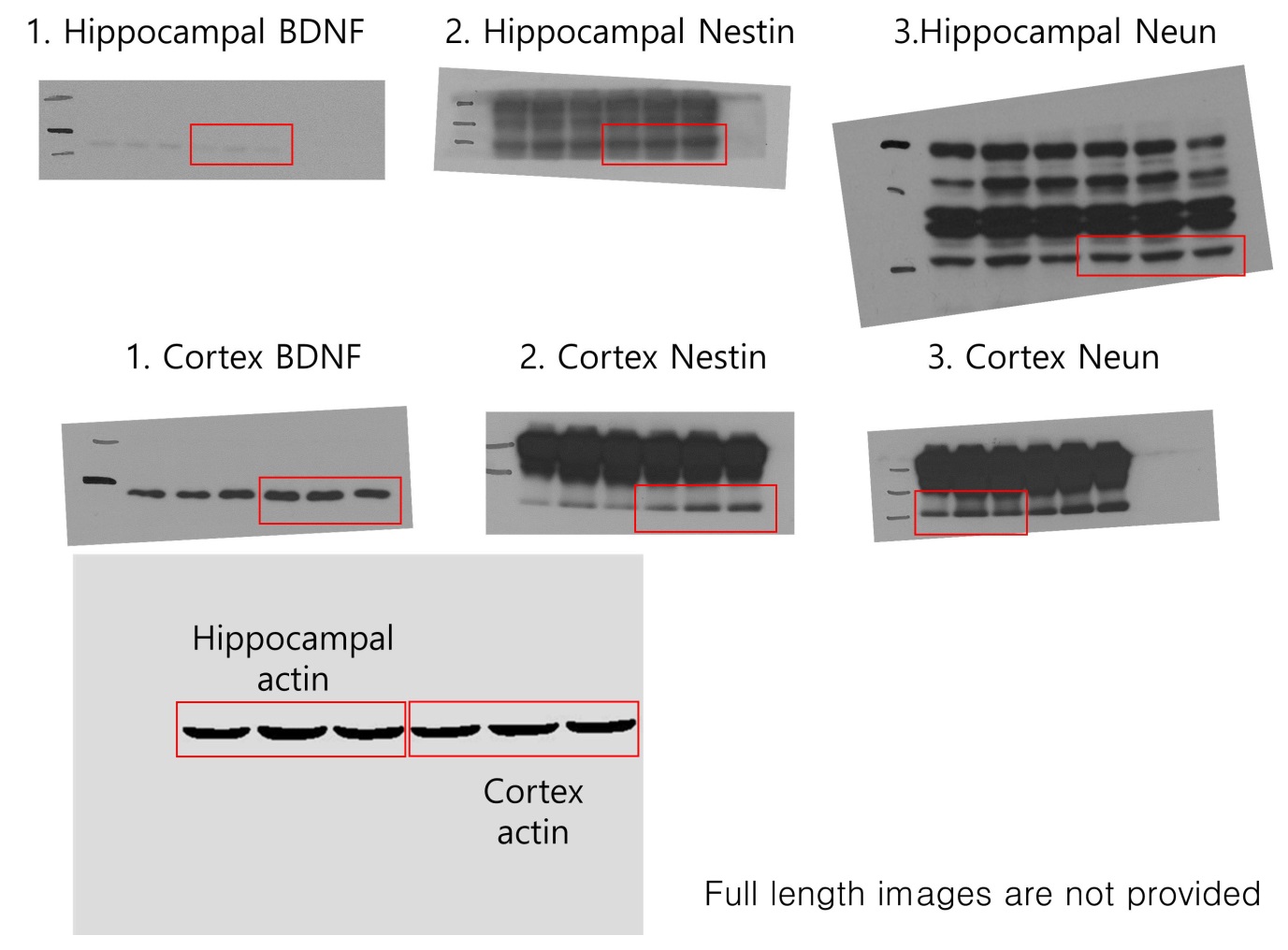


**Fig.S3. Supplementary gel data for fig. 4-actin contrast**

**
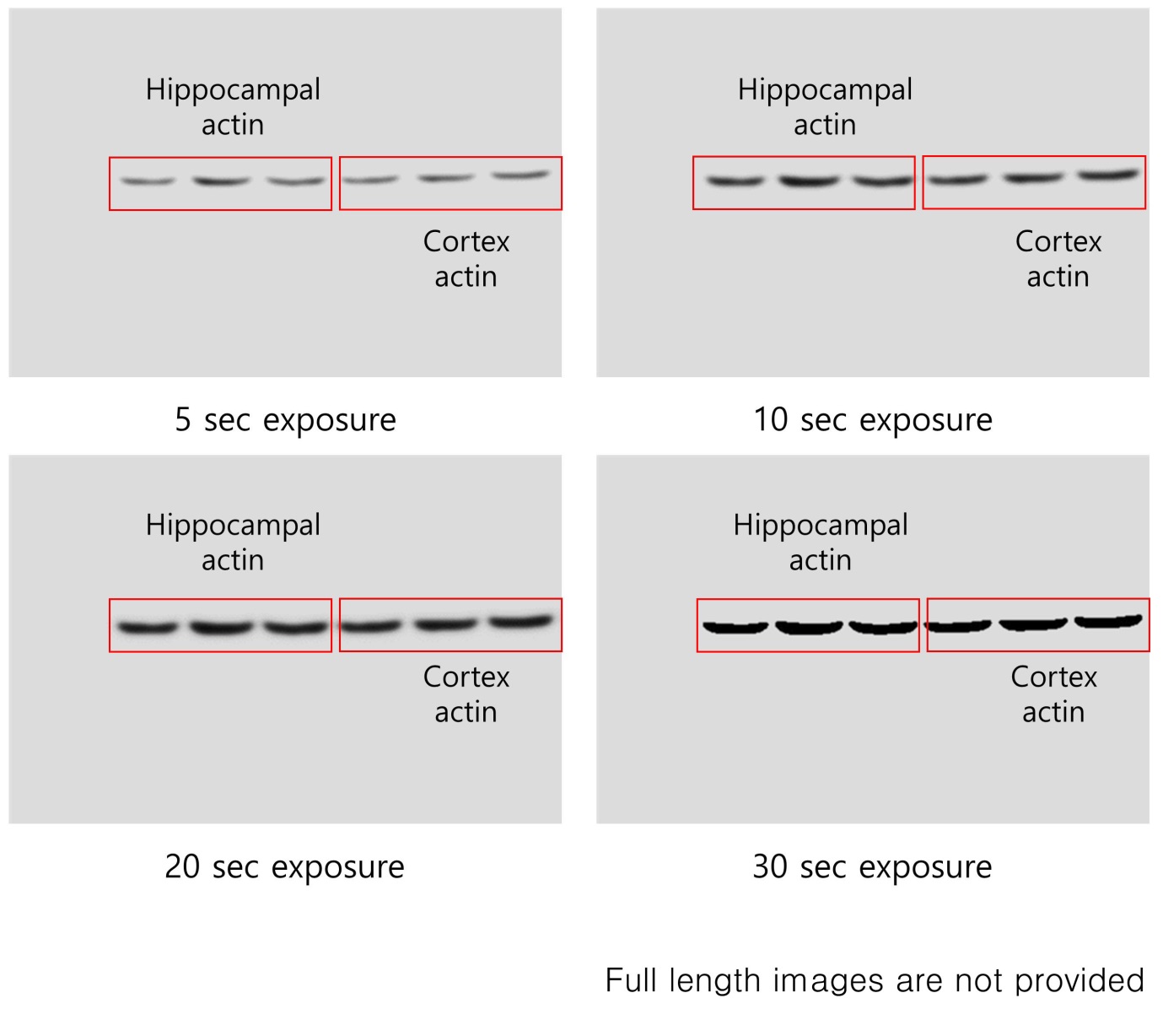
**

**Fig.S4. Supplementary gel data for fig. 5**

**
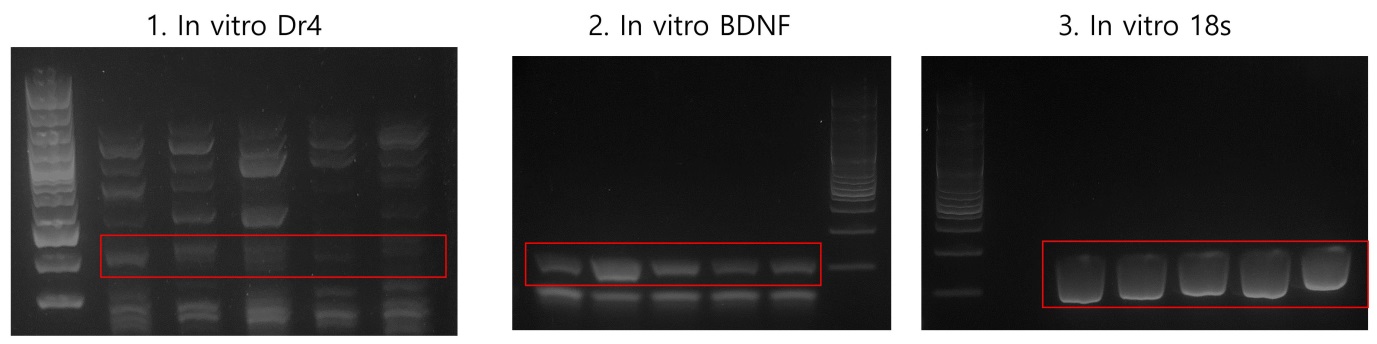
**
